# Supplementary material for: CAM: A quality control pipeline for MNase-seq data
Source: PLoS One. 2017 Aug 7;12(8):e0182771. doi: 10.1371/journal.pone.0182771 (PMC5546596; doi:10.1371/journal.pone.0182771)
Supplement: S1 File — (PDF) [file pone.0182771.s001.pdf]

QC and analysis reports for MNase-seq data : GSM907784

May 19, 2017

Contents

1 Data description **2**

2 QC measurements **3**

2.1 Sequencing coverage 3

2.2 AA/TT/AT di-nucleotide frequency 4

2.3 Nucleosomal DNA length distribution 5

2.4 Nucleosome profile on potentially funcitonal regions 6

2.5 Nucleosome free regions (NFR) and nucleosome positioning on promoters 7

2.6 Well-positioned nucleosome arrays 8

3 Output list **9**

1 Data description

Table 1 mainly describes the input file and parameters for the CAM pipeline.

Table 1: Data description

Parameter	Value
output name	GSM907784
input file A	GSM907784.bed
input file B	NA
input format	BED
sequencing type	Paired end
genome version (species)	hg19
Q30 filter mapped reads	True
custom region	CTCF_motif_hg19.bed

2 QC measurements

We provided multiple key measurements: 1) sequencing coverage, 2) AA/TT/AT di-nucleotide frequency, 3) nucleosomal DNA length, 4) existence of nucleosome free regions (NFR) at promoters, 5) well-positioned nucleosomes at downstream promoters, 6) well-positioned nucleosomes at custom defined potential cis-regulatory regions, 7) enrichment of well-positioned nucleosome arrays in DNase hypersensitive sites (DHS)

2.1 Sequencing coverage

Sequencing coverage provides a direct measurement of the resolution of two features of nucleosome organization, i.e. occupancy and positioning (Struhl and Segal, 2013). Sequencing coverage is defined as: (Number of reads * 194bp)/(Effective genome size). "Number of reads" is the number of mappable reads after MAPQ filtering. "194bp" is the total length of nucleosomal DNA and linker DNA estimated from historical data. "Effective genome size" is defined as 2.7e9 bp for human and 1.87e9 bps for mouse. Below we plotted the distribution of sequencing coverage of historical data; the sequencing coverage of the input data was marked by vertical line: 39.14.

Figure 1: Sequencing coverage

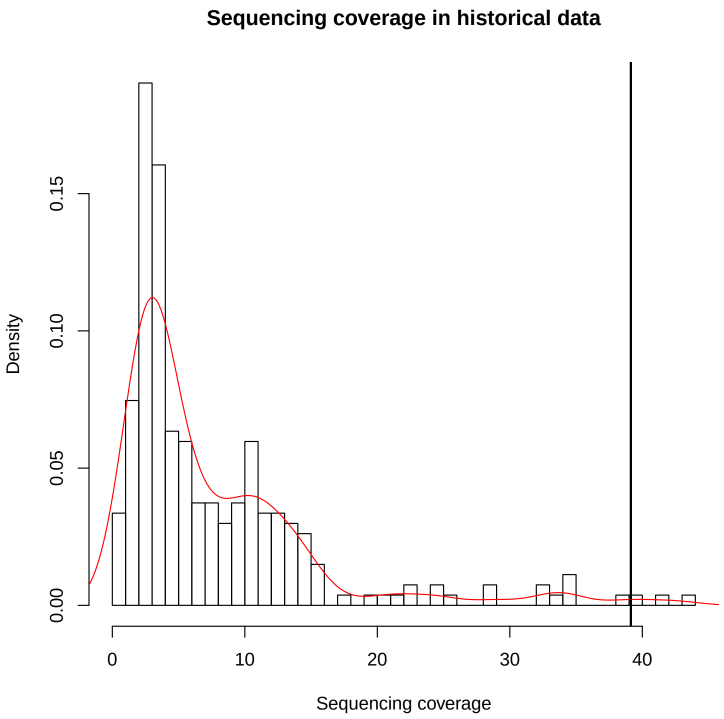

2.2 AA/TT/AT di-nucleotide frequency

The 10-base AA/TT/AT periodicity in nucleosomal DNA provides a measurement of nucleosome rotational positioning, which has been shown to be influenced by DNA sequence (Satchwell, et al., 1986). Mappable reads were sampled down to 10 million and were extended to 147 bp in their 3'end direction. Then the aggregate AA/TT/AT di-nucleotide frequency across 4th - 143th bp of the extended reads was calculated (right). We conducted a Fourier transform on the aggregate frequency and used the energy of 10-bp periodicity (defined as rotational score) to show the extent the MNase-seq reads reflect nucleosome organization. Samples with rotational score greater than 0.08 were defined as "Pass" in this measurement, otherwise they were defined as "Fail". The cutoff 0.08 was determined by the distribution of rotational scores from all historical data (left, vertical line marked the rotational score of the input sample: 0.2957 [Pass]).

Figure 2: AA/TT/AT di-nucleotide frequency

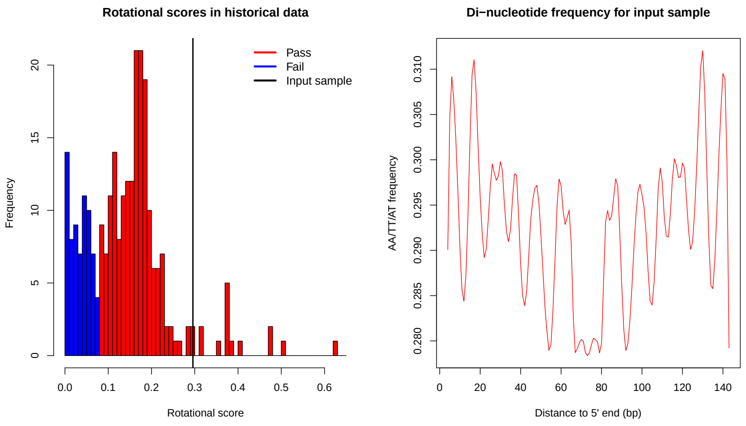

2.3 Nucleosomal DNA length distribution

Nucleosomal DNA length distribution (refer to fragment length or MNase library size) is closely related and thus can reflect the degree of MNase digestion. For paired end sample, fragment length distribution from all mappable fragments was used directly to infer the nucleosomal DNA length distribution. For single end sample, we calculated a start-to-end distance to estimate the nucleosome length: mappable reads were sampled down to 10 million and then we calculated the distribution of the distance from 5'end of each plus strand read to all 5'end of minus strand reads within 250 bp downstream. Duplicate reads were discarded in this calculation. After the distribution of nucleosomal DNA length was generated, the length with the highest frequency was defined as the estimated nucleosomal DNA length of the input sample (left, for both paired end and single end data). Samples with nucleosomal DNA length within 140bp - 155bp were defined as "Pass", otherwise they were defined as "Fail". The cutoff was determined by the distribution of nucleosomal DNA length from all historical data (left, vertical line marked the nucleosomal DNA length of the input sample: 149 [Pass]).

Figure 3: Nucleosomal DNA length distribution

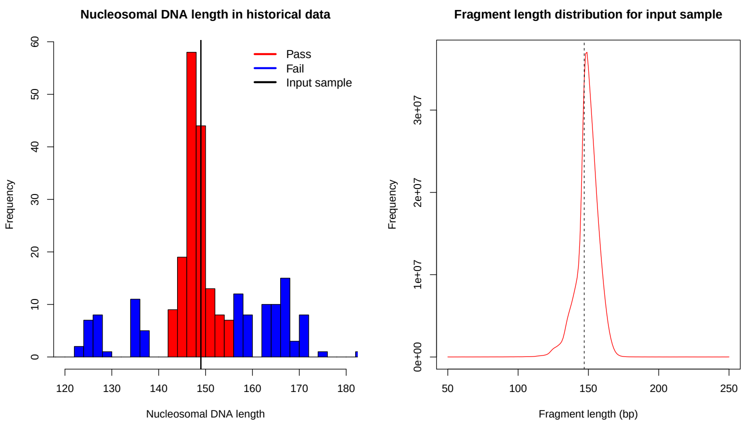

2.4 Nucleosome profile on potentially funcitonal regions

CAM generated the aggregate curve and the heatmap of nucleosome organization on promoter regions and the custom regions: CTCF_motif_hg19.bed in 10 bp resolution. Signal from minus strand regions were reversed in both heatmap and aggregate curve. The signal for each region was also outputted as matrix: GSM907784_Tss_profile.txt & GSM907784_CustomRegion_profile.txt.

Figure 4: Nucleosome profile on potentially funcitonal regions

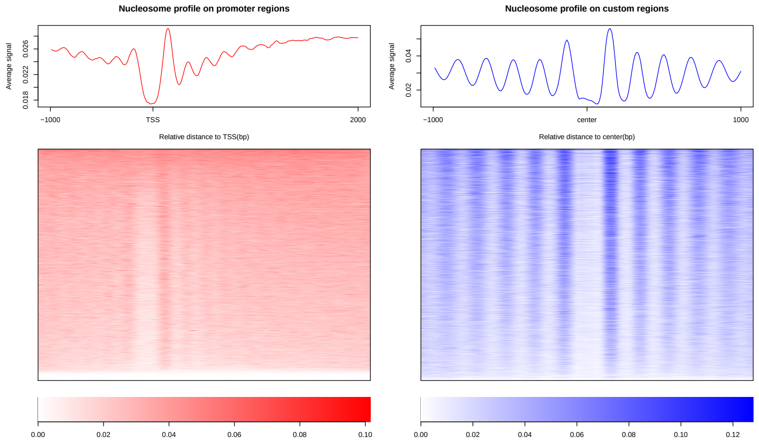

2.5 Nucleosome free regions (NFR) and nucleosome positioning on promoters

Based on nucleosome profile on promoters, CAM generated two scores to describe the nucleosome positioning on promoters. First, promoter NFR scores described the fold change of the MNase-seq signal of nucleosome free regions compared to the +1 nucleosome and -1 nucleosome. The higher the promoter NFR score is, the deeper the nucleosome free region is. Lower promoter NFR score associated with weak or none nucleosome free regions, which may indicate reads from open chromatins. Samples with promoter NFR scores higher than 0.4 were defined as "Pass", otherwise they were defined as "Fail".The cutoff was determined by the distribution of NFR scores from all historical data (left, vertical line marked the promoter NFR score of the input sample: 0.8823 [Pass]). Next, promoter nucleosome positioning score defined whether clear nucleosome positioning pattern was observed from downstream promoters. The promoter nucleosome positioning score was calculated by the coefficient of variance (CV) of the distance between the +1, +2, +3 and +4 nucleosomes. The lower the nucleosome positioning score is, the better nucleosome positioning was observed on downstream promoters. Samples with nucleosome positioning scores lower than 0.4 was defined as "Pass", otherwise they were defined as "Fail".The cutoff was determined by the distribution of nucleosome positioning scores from all historical data (right, vertical line marked the positioning score of the input sample: 0.0833 [Pass]).

Figure 5: NFR and nucleosome positioning on promoters

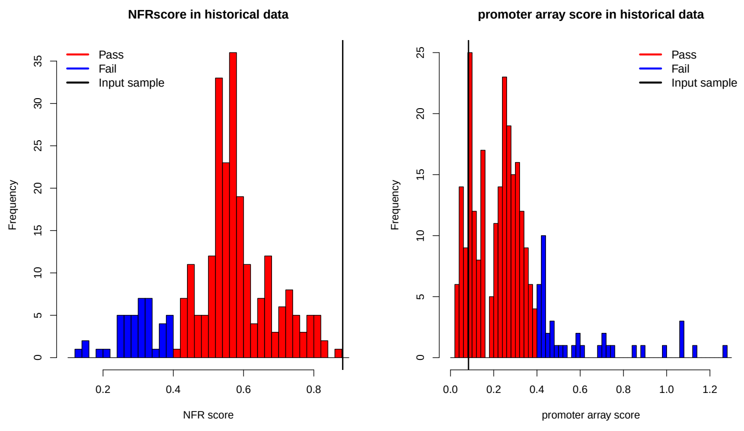

2.6 Well-positioned nucleosome arrays

Regions with well-positioned nucleosome arrays were detected as previous described (Zhang, et al., 2014), and the enrichment in potential regulatory regions (downstream promoters and the union DNase I hypersensitive sites (DHS)) was listed. Enrichment was defined as observed/expected percentage of nucleosome array on promoters (>1 for enriched). Expected percentage was equal to the percentage of promoter length compared to the total length of effective genome. Similar approach was applied on the union DHS sites. For each region with well-positioned nucleosome array, its genomic coordinates together with the nucleosome profile value were reported in the output file: profile on Nucleosome Array. Samples with fold enrichment of DHS sites less than 2 were regarded as Fail in this measurement, indicating the well-positioned nucleosome arrays are more likely to be caused by random rather than the barrier nucleosome model. The gene level annotation of nucleosome arrays was also reported in output file: geneLevel nucarrayAnnotation (5th column represents overlapped nuc-array length)

Table 2: Enrichment of well-positioned nucleosome arrays

Genomic region(Category)	Enrichment
downstream promoter	2.1149
union DHS sites	4.0039 [Pass]

3 Output list

All output files were described in the following table.

Table 3: output list

Filename	Description
GSM907784_profile.bw	genome-wide nucleosome profile
GSM907784_Tss_profile.txt	nucleosome signal on promoter regions
GSM907784_CustomRegion_profile.txt	nucleosome signal on custom regions
GSM907784_Nucleosome_Array.bed	well-positioned nucleosome arrays
GSM907784_geneLevel_nucarrayAnnotation.bed	gene level annotation of nucleosome arrays
GSM907784_profile_on_Nucleosome_Array.bw	nucleosome signal on well-positioned nucleosome arrays
GSM907784_summary.pdf	summary QC report
